# SARS-CoV-2 Spike Protein Is Capable of Inducing Cell–Cell Fusions Independent from Its Receptor ACE2 and This Activity Can Be Impaired by Furin Inhibitors or a Subset of Monoclonal Antibodies

**DOI:** 10.3390/v15071500

**Published:** 2023-07-04

**Authors:** Nina Reuter, Xiaohan Chen, Barbara Kropff, Antonia Sophia Peter, William J. Britt, Michael Mach, Klaus Überla, Marco Thomas

**Affiliations:** 1 Virologisches Institut, Klinische und Molekulare Virologie, Friedrich-Alexander-Universität Erlangen-Nürnberg, 91054 Erlangen, Germany; nina.reuter@uk-erlangen.de (N.R.); xiaohan.chen@uk-erlangen.de (X.C.); barbara.kropff@uk-erlangen.de (B.K.); antoniasophia.peter@uk-erlangen.de (A.S.P.); michael.mach@fau.de (M.M.); klaus.ueberla@fau.de (K.Ü.); 2 Departments of Pediatrics, Microbiology and Neurobiology, Children’s Hospital of Alabama, School of Medicine, University of Alabama, Birmingham, AL 35233-1771, USA; wbritt@uabmc.edu

**Keywords:** severe acute respiratory syndrome coronavirus 2 (SARS-CoV-2), glycoprotein, spike, variants of concern (VOCs), ACE2, receptor-independent, cell–cell fusion, screening, therapy, neutralization, antibodies, furin, inhibitors

## Abstract

Severe acute respiratory syndrome coronavirus 2 (SARS-CoV-2), which was responsible for the COVID-19 pandemic, efficiently spreads cell-to-cell through mechanisms facilitated by its membrane glycoprotein spike. We established a dual split protein (DSP) assay based on the complementation of GFP and luciferase to quantify the fusogenic activity of the SARS-CoV-2 spike protein. We provide several lines of evidence that the spike protein of SARS-CoV-2, but not SARS-CoV-1, induced cell–cell fusion even in the absence of its receptor, angiotensin-converting enzyme 2 (ACE2). This poorly described ACE2-independent cell fusion activity of the spike protein was strictly dependent on the proteasomal cleavage of the spike by furin while TMPRSS2 was dispensable. Previous and current variants of concern (VOCs) differed significantly in their fusogenicity. The Delta spike was extremely potent compared to Alpha, Beta, Gamma and Kappa, while the Omicron spike was almost devoid of receptor-independent fusion activity. Nonetheless, for all analyzed variants, cell fusion was dependent on furin cleavage and could be pharmacologically inhibited with CMK. Mapping studies revealed that amino acids 652-1273 conferred the ACE2-independent fusion activity of the spike. Unexpectedly, residues proximal to the furin cleavage site were not of major relevance, whereas residue 655 critically regulated fusion. Finally, we found that the spike’s fusion activity in the absence of ACE2 could be inhibited by antibodies directed against its N-terminal domain (NTD) but not by antibodies targeting its receptor-binding domain (RBD). In conclusion, our BSL-1-compatible DSP assay allowed us to screen for inhibitors or antibodies that interfere with the spike’s fusogenic activity and may therefore contribute to both rational vaccine design and development of novel treatment options against SARS-CoV-2.

## 1. Introduction

Severe acute respiratory syndrome coronavirus 2 (SARS-CoV-2), the causative agent of the global COVID-19 pandemic, is a novel beta-coronavirus that is closely related to two other highly pathogenic human coronaviruses, SARS-CoV-1 and MERS-CoV [1,2,3]. SARS-CoV-2 is an enveloped, positive-sense single-stranded RNA virus that enters the host cell by fusing its lipid bilayer with the target cell membrane. Similar to SARS-CoV-1, SARS-CoV-2 uses the highly glycosylated, homotrimeric spike protein, a class I viral fusion protein, for entry [4,5,6]. The spike protein is produced as a single-chain precursor and processed in the virus-producer cell by host proprotein convertases such as furin into the non-covalently associated receptor-binding subunit S1 and the fusion fragment S2 [7,8]. In contrast to SARS-CoV-1, the spike protein of SARS-CoV-2 contains a polybasic R-X-X-R cleavage motif for furin-like enzymes at the S1/S2 boundary [9]. The S1 subunit harboring the receptor-binding domain (RBD) binds to the host angiotensin-converting enzyme 2 (ACE2) [10,11]. After engagement with the ACE2 receptor, an additional site inside the spike’s S2 subunit is exposed, referred to as S2′ [6]. Cleavage of S2′ by the host transmembrane serine protease 2 (TMPRSS2) at the plasma membrane or by Cathepsin L in the endosomal compartment following ACE2-mediated endocytosis triggers a cascade of S2 refolding events resulting in the release of the hydrophobic fusion peptide (FP), thereby facilitating membrane fusion [12,13,14]. Thus, both proteolytic cleavage events at the multibasic S1–S2 junction and the S2′ site play a critical role in initiating the membrane fusion process [6,15] and can be blocked by furin (decanoyl-RVKR-chloromethylketone, CMK) and TMPRSS2 inhibitors (nafamostat or camostat), respectively [16,17].

Apart from the entry of cell-free viral particles, SARS-CoV-2 has been shown to preferentially spread in cultured cells and tissues via cell-to-cell transmission [18]. In SARS-CoV-2-infected cells, the spike protein localized to the plasma membrane is capable of inducing cell–cell fusion with neighboring uninfected cells, which can lead to the formation of enlarged multinucleated giant cells referred to as syncytia [19,20]. The presence of SARS-CoV-2-infected syncytial pneumocytes as well as heterocellular syncytia containing lymphocytes has been reported in postmortem lung samples of patients with severe COVID-19, demonstrating the physiological relevance of spike-driven syncytium formation [21,22,23]. Hence, the ability of the spike to induce cell–cell fusions and syncytium formation provides an additional mechanism for SARS-CoV-2 dissemination in vivo, thereby contributing to viral pathology.

Since the emergence of the ancestral Wuhan-Hu-1 (WT) strain, several variants of concern (VOCs) have become globally dominant, including Alpha (B.1.1.7), Beta (B1.351), Gamma (P.1 and P.2), Delta (B1.617.2) and Omicron (B.1.1.529), which has led to the selection of mutations in the spike protein. Studies have shown that emerging VOCs with increased pathogenicity are associated with spike polymorphisms, which are characterized by an enhanced capacity for both fusion activity and syncytia formation [24,25]. Thus, changes in the spike protein affecting its fusion activity appear to significantly impact SARS-CoV-2 virulence and pathogenicity. 

In order to dissect the cell membrane fusion activity of the spike in further detail, we established a dual split protein (DSP) assay using GFP and luciferase as reporter systems to quantify cell–cell fusions. In this report, we provide several lines of evidence that the spike protein is capable of inducing cell–cell fusion in an ACE2 receptor-independent manner. (i) Considerable cell fusion activity of the spike protein was observed in ACE2-negative HEK293T, which otherwise cannot be infected with spike pseudotyped lentiviral particles or authentic virus. (ii) Although both proteolytic cleavage sites S1/S2 and S2′ were of importance for the ACE2-independent fusion activity of the spike, cell–cell fusion could only be blocked with inhibitors of furin but not by TMPRSS2 inhibitors. (iii) Monoclonal antibodies (mAbs) targeting the RBD of the spike, thereby neutralizing viral infections, failed to block spike-induced fusions in the absence of ACE2, while mAbs targeting the NTD effectively prevented receptor-independent fusions.

## 2. Materials and Methods

### 2.1. Oligonucleotides and Expression Constructs

Oligonucleotides used in this study for cloning or site-directed mutagenesis were purchased from biomers.net GmbH (Ulm, Germany) and are listed in Table 1. 

The dual split protein expression vectors pCMV-DSP1-7 (pE85) and pCMV-DSP8-11 (pE86) were kindly provided by Zene Matsuda (Research Center for Asian Infectious Diseases, Institute of Medical Science, University of Tokyo, Tokyo, Japan) [26]. For lentiviral transduction, the DSP1-7 and DSP8-11 coding sequences were cloned into a pCDH-EF1-based retroviral vector generating lentiviral constructs designated as pMN350 and pMN351. The expression plasmids coding for HCMV gB and the chimera gB/VSV-G were described previously [27]. The expression construct for avian influenza virus hemagglutinin of strain H7N7 (H7N7 HA) was a kind gift of Matthias Tenbusch (Institute of Clinical and Molecular Virology, Erlangen, Germany), while the expression vectors for HIV-1 gp160, the SARS-CoV-1 spike and the proprotein convertase furin were kindly provided by Klaus Überla (Institute of Clinical and Molecular Virology, Erlangen, Germany). The SARS-CoV-2 spike variants Wuhan-Hu-1 (WT), Alpha, Beta, Gamma, Kappa, Delta and Omicron were obtained by cloning the respective coding sequences into the eukaryotic expression vector pCG1. The human ACE2 construct was generated by cloning a respective PCR product obtained with primers 2-58 and 2-59 into a pcDNA3.1 expression plasmid. The following plasmid expression constructs were generated through site-directed mutagenesis: the WT (Wuhan-Hu-1) spike proteolytic cleavage mutants S1mut and S2′mut as well as the double mutant S1+S2′mut using primer pairs 3-71 and 3-72 and/or 3-73 and 3-74, respectively; the WT spike pre-lock mutant with primers 3-91 and 3-92; the dominant-negative furin mutant DN-Furin using primers 4-11 and 4-12; the spike mutants Delta K+H and Delta H655Y with primers 4-20 and 4-21 or 4-30 and 4-31, respectively; the spike mutants Omicron N+R, Omicron Y655H and Omicron H+N+R using primer pairs 4-34 and 4-35 and/or 4-28 and 4-29, respectively. The Delta-Omicron chimeras Del-Om and Om-Del were generated by cut and paste cloning using the restriction enzymes PflMI and XbaI (New England Biolabs, Ipswich, MA, USA). The integrity of all newly generated plasmids was confirmed through automated DNA sequence analysis (Macrogen, Amsterdam, Netherlands).

### 2.2. Cell Culture

Human embryonic kidney cells (HEK293T) were cultured in Dulbecco’s modified Eagle’s medium (DMEM) (Gibco, Thermo Fisher Scientific, Waltham, MA, USA) supplemented with 10% fetal calf serum (FCS) (Sigma-Aldrich, St. Louis, MO, USA), glutamine (100 μg/mL), and gentamicin (350 μg/mL).

### 2.3. Generation of 293T-DSP Cells through Lentivirus Transduction

For the generation of the 293T-DSP1-7 and 293T-DSP8-11 cells stably expressing the chimeric reporter proteins composed of split Renilla luciferase (RLucN and RLucC) and split GFP (GFP1-7 and GFP8-11), replication-deficient lentiviruses were generated using the pCDH-EF1α-based expression vectors pMN350 and pMN351, respectively. To this end, 5 × 10^6^ HEK293T cells seeded in 10 cm dishes were transfected with either pMN350 or pMN351 together with packaging plasmids psPAX2 and pLP/VSV-G using the Lipofectamine 2000 reagent (Invitrogen, Life Technologies, Carlsbad, CA, USA). One day after transfection, cells were provided with fresh medium. At 48 h post-transfection, viral supernatants were harvested, cleared through centrifugation, filtered using a 0.45 µm sterile filter and stored at −80 °C. Thereafter, HEK293T cells were incubated for 24 h with lentivirus supernatant in the presence of 10 µg/mL Polybrene (Sigma-Aldrich, Merck KGaA, Darmstadt, Germany). The stably transduced cell populations 293T-DSP1-7 and 293T-DSP8-11 were assessed for correct expression of the chimeric reporter proteins through transfection of the respective split counterpart expression constructs pE85 and pE86, respectively. Finally, the generated 293T-DSP1-7 and 293T-DSP8-11 cells were co-cultured in a 1:1 ratio to obtain the 293T-DSP-mix cells.

### 2.4. Dual Split Protein Cell Membrane Fusion Assay (DSP Assay)

293T-DSP-mix cells seeded in 24-well (2 × 10^5^ cells/well) or 96-well plates (2.5 × 10^4^ cells/well) were transfected with the indicated viral fusion protein expression constructs through calcium phosphate precipitation. At 4 h post-transfection, cells were washed with phosphate-buffered saline (PBS) and incubated with fresh medium containing monoclonal antibody preparations or inhibitory substances as indicated. For the detection of GFP signals, cells were imaged three days post-transfection with a CTL Immunospot^®^S6 UV Analyzer (Cellular Technology Limited, Bonn, Germany) and GFP counts determined using the ImmunoSpot 6.0.0.2 software version (Cellular Technology Limited). For luminescence measurement, cells seeded in ViewPlate-96 black dishes (PerkinElmer, Waltham, MA, USA) were treated 24–48 h post-transfection with 60 µM of the membrane-permeable RLuc substrate EnduRen (Promega, Madison, WI, USA). At 2–24 h after the addition of the live cell substrate EnduRen, RLuc activity was measured by using an Orion microplate lumino-meter (Berthold Technologies, Bad Wildbach, Germany). Values for GFP counts or RLU were derived minimally from biological triplicates and represent mean values ± standard deviations. For all DSP cell fusion assays, Western blot experiments were performed in parallel to confirm similar expression levels of the viral fusion proteins by harvesting and lysing the transfected 293-DSP-mix cells after GFP and luciferase signal measurement as detailed in the immunoblotting section of Materials and Methods.

### 2.5. Pseudovirus Entry Assay

Lentiviruses pseudotyped with the SARS-CoV-2 spike were generated in HEK293T cells as described previously [28,29]. Briefly, HEK293T cells were co-transfected with a SIV-based self-inactivating vector encoding luciferase (pGAE-LucW), the SIV-based packaging plasmid (pAdSIV3), and a WT spike-encoding plasmid. Pseudoviruses were harvested 72 h after transfection and used for the infection of 293T-DSP-mix cells transfected with an ACE2 expression construct or empty vector (mock control). At 48 hpi, cells were lysed through the addition of 100 µL Bright Glo lysis buffer (Promega) for 15 min at 37 °C. Three minutes later, after the addition of 25 µL Bright Glo substrate (Promega), the luciferase signals were measured using a microplate luminometer (VICTOR X5, PerkinElmer) and the PerkingElmer 2030 Manager software.

### 2.6. Indirect Immunofluorescence Analysis

For indirect immunofluorescence analysis, 293T-DSP-mix cells grown on coverslips were transfected via calcium phosphate precipitation. At three days post-transfection, cells were washed twice with PBS and fixed with a 4% paraformaldehyde solution for 10 min at room temperature. After washing three times with PBS, cells were permeabilized with PBS-0.2% Triton X-100 on ice for 20 min. Cells were washed again four times with PBS and blocked in PBS-1% bovine serum albumin (BSA) at room temperature for 30 min. After incubation with the respective primary and secondary antibodies for 45 min at 37 °C, coverslips were mounted onto microscope slides using DAPI (4′,6-diamidino-2-phenylindole)-containing Vectashield mounting medium (Vector Laboratories, Newark, CA, USA). The samples were imaged with a Leica TCS SP5 confocal microscope with 488 nm and 543 nm laser lines, scanning each channel separately under image capture conditions that eliminated channel overlap. The images were exported, processed with Adobe Photoshop Elements 15 and assembled by using CorelDraw X6.

### 2.7. Immunoblotting

Lysates from transfected 293T-DSP-mix cells were prepared in sodium dodecyl sulfate polyacrylamide gel electrophoresis (SDS-PAGE) loading buffer, boiled at 95 °C for 10 min and sonicated for 1 min. Proteins were separated through SDS-PAGE using 12.5% polyacrylamide gels followed by transfer onto nitrocellulose blotting membranes (Cytivia, Amersham Biosciences Europe GmbH, Freiburg, Germany). Chemiluminescence detection was performed according to the manufacturer’s protocol (ECL Western blotting detection kit; Amersham Biosciences Europe GmbH) by using an INTAS advanced fluorescence imager (INTAS Science Imaging Instruments GmbH, Göttingen, Germany).

### 2.8. Antibodies

The following antibodies directed against the SARS-CoV-2 spike were used: neutralizing antibodies with murine Fc-portions recognizing either the NTD (TRES328, TRES544, TRES631, TRES1209, TRES1293, TRES618, TRES49 and TRES219) or the RBD (TRES567, TRES6 and TRES224) of the spike protein as well as the non-neutralizing anti-spike antibodies TRES208, TRES468, TRES43.3 and TRES480 [30], the human mAb 4C12 (kindly provided by Garvan Institute of Medical Research, Sydney, Australia, unpublished data Stahl-Hennig, Peter et al., WO2022133545A1, [31]) and the rabbit polyclonal antibody NB100-56578 (Novus Biologicals, Wiesbaden, Germany). The Alexa Fluor 488- and 555-conjugated secondary antibodies for indirect immunofluorescence analyses were purchased from Invitrogen (Thermo Fisher Scientific, Waltham, MA, USA). The horseradish peroxidase-conjugated secondary antibodies for Western blotting were obtained from Dako (Agilent Technologies, Santa Clara, CA, USA). The monoclonal beta-actin antibody AC-15 was used for control reactions (Sigma-Aldrich, St. Louis, MO, USA).

## 3. Results

### 3.1. Establishment of a Dual Split Protein (DSP) Assay for Automated Quantification of Cell–Cell Fusions

**Figure 1:**
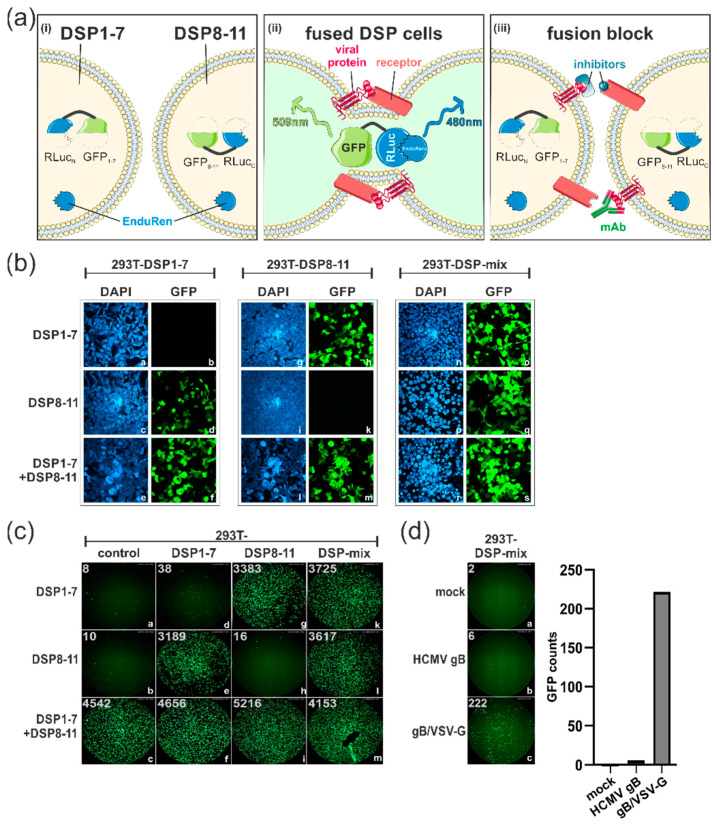
Dual split protein (DSP) assay for the automated quantification of cell–cell fusion. (**a**) Schematic representation of the DSP assay. (i) HEK293T cells were transduced with lentiviruses coding for split chimeric reporter proteins composed of GFP and Renilla Luciferase (RLuc), resulting in DSP1-7 and DSP8-11 cells, which were mixed in a 1:1 ratio. (ii) Co-cultured DSP cells were transfected with plasmids encoding diverse viral fusion proteins either alone or in combination with a plasmid coding for their corresponding receptor. Membrane fusion was quantified through detection of GFP-fluorescence and/or bioluminescence upon RLuc-mediated cleavage of its cell-permeable substrate EnduRen. (iii) The DSP assay was used to identify monoclonal antibodies and inhibitors that block viral glycoprotein-mediated cell–cell fusions. This figure was generated using templates from SMART Servier Medical Art By Servier, used under CC BY 3.0, https://smart.servier.com/, accessed on 1 June 2022. (**b**) Immunofluorescence analyses of lentivirally transduced HEK293T cells 293T-DSP1-7, 293T-DSP8-11 and 293T-DSP-mix. The individual DSP cell cultures were transfected with pcDNA3-based vectors encoding DSP1-7 (panels a and b), DSP8-11 (panels c and d) or a combination of both (DSP1-7 + DSP8-11; panels e and f). Two days later, cells were fixed, cell nuclei visualized through DAPI and the GFP-fluorescence of the reconstituted DSP reporter protein examined through confocal laser scanning microscopy. (**c**) Automated quantification of the GFP counts obtained after transfection of the respective DSP cells (293T-DSP1-7, -DSP8-11 and -DSP-mix) with the indicated DSP expression constructs using a CTL-Fluorospot reader. The numbers in the left corner of each image represent the GFP counts of each well as calculated using the software ImmunoSpot (version 6.0.0.2). (**d**) Validation of the assay via transfection of 293T-DSP-mix cells with gB of human cytomegalovirus (HCMV) (panel b) or the intrinsically fusion-active derivative gB/VSV-G (panel c). The GFP counts measured using the Fluorospot reader (upper left corner of the immunofluorescence images) are diagramed as a column chart.

The cell–cell fusion activity of the spike has been shown to contribute to efficient cell-to-cell transmission of SARS-CoV-2 [18]. In order to quantify the membrane fusion capacity of viral glycoproteins, we established a dual split protein (DSP) fusion assay. As illustrated in the schematic representation of Figure 1a, this assay is based on a pair of chimeric reporter proteins composed of split Renilla luciferase (RLucN and RLucC) and split GFP (GFP1-7 and GFP8-11) (Figure 1a, (i)). Following lentiviral gene transfer, HEK293T cells stably expressing the respective dual split proteins were generated (termed DSP1-7 and DSP8-11) and co-cultured in a 1:1 ratio (Figure 1a, (i)). After transfection of the mixed cells, the expressed viral glycoproteins can bind to their cognate receptor to induce membrane fusions. This pore formation leads to content mixing of the cells, re-association of the dual split reporter and recovery of the GFP-fluorescence and RLuc activity, which can be measured upon cleavage of the cell-permeable substrate EnduRen (Figure 1a, (ii)). Finally, the DSP assay permits the screening and identification of small molecule inhibitors or monoclonal antibodies (mAbs) that can block virus glycoprotein-induced membrane fusion (Figure 1a, (iii)). Initially, the successful generation of HEK293T-DSP cells was verified using immunofluorescence (Figure 1b). For this, 293T-DSP1-7 (Figure 1b, panels a–f) and 293T-DSP8-11 (Figure 1b, panels g–m), either alone or co-seeded in a 1:1 ratio and hereafter referred to as 293T-DSP-mix (Figure 1b, panels n–s), were transfected with expression constructs coding for either DSP1-7 or DSP8-11. As expected, GFP-positive cells were only detectable upon expression of the respective DSP counterparts (Figure 1b, panels d, h, o and q) or upon co-expression of both DSP plasmids (Figure 1b, panels f, m and s), confirming that split reporter molecules remained inactive (Figure 1b, panels b and k). These results were confirmed by using a Fluorospot reader for automated quantification of the number of green fluorescent cells (Figure 1c). The measured GFP counts of each well are given in the upper left corner of images a–m; Figure 1c. Finally, to test whether the DSP assay could be utilized to measure cell–cell fusions induced by viral fusion proteins, the 293T-DSP-mix cells were transfected with plasmids encoding human cytomegalovirus (HCMV) glycoprotein B (gB) as well as its derivative gB/VSV-G, a chimer consisting of gB and its structurally related class III viral fusion protein G of the vesicular stomatitis virus (VSV-G) (Figure 1d). In previous studies, we demonstrated that gB is generally non-fusogenic when expressed by itself [27]. However, fusion of the ectodomain of gB to the transmembrane and carboxyterminal domain of VSV-G resulted in an intrinsically fusion-active variant, gB/VSV-G [27]. In agreement with our previous findings, we found that the expression of gB/VSV-G resulted in over 200 GFP counts compared to fusion-negative native gB, which behaved like the mock control (Figure 1d). Taken together, these results confirmed the successful establishment of an experimental system for automated quantification of cell–cell fusions induced by viral glycoproteins.

### 3.2. SARS-CoV-2 Spike Protein Can Induce ACE2-Independent Cell–Cell Fusions

**Figure 2:**
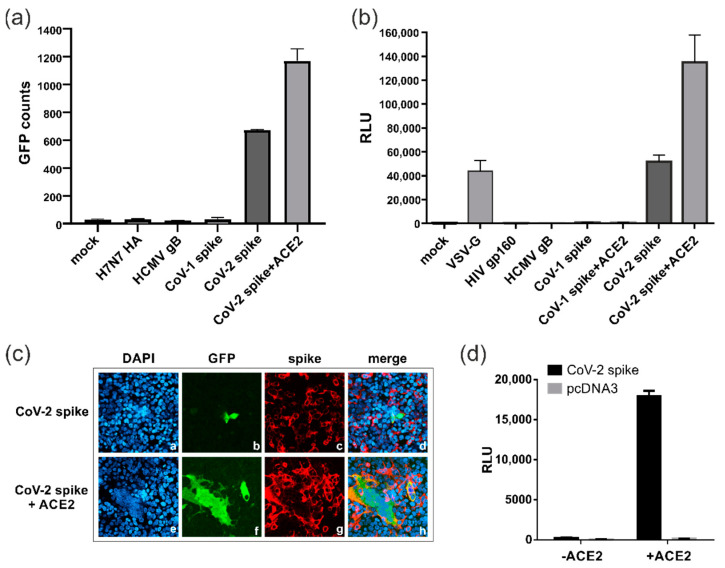
Cell fusion activity of SARS-CoV-2 spike protein is not dependent on ACE2 expression. (**a**,**b**) The 293T-DSP-mix cells were transfected with vectors encoding the indicated viral fusion proteins or an empty vector (mock). Cell–cell fusions were quantified (**a**) via GFP using the CTL-Fluorospot reader or (**b**) via bioluminescence, given in relative light units (RLU) upon RLuc-mediated cleavage of EnduRen. (**c**) Immunofluorescence analyses of 293T-DSP-mix cells transfected with SARS-CoV-2 spike alone (panels a–d) or in combination with its receptor ACE2 (panels e–h). Three days later, cells were fixed, cell nuclei visualized using DAPI and the GFP-signal of the reconstituted DSP-reporter protein (panels b and f) examined through confocal laser scanning microscopy. As a control, spike expression was confirmed by using mAb TRES567 (panels c and g). (**d**) Pseudovirus entry assay using SARS-CoV-2 spike (CoV-2 spike) or mock (pcDNA3) pseudotyped lentiviral particles for infection of 293T-DSP-mix cells, which were either transfected with (+ACE2) or without ACE2 (-ACE2). Two days post-infection, luciferase reporter gene activity was measured, and this is given in RLU.

In the next step, we applied the DSP assay to evaluate the fusion activity of the SARS-CoV-2 (CoV-2) spike (Wuhan-Hu-1) compared to other viral fusion proteins, including the spike of SARS-CoV-1 (CoV-1), the causative agent of the SARS outbreak of 2002/2003 (Figure 2). To this end, the 293T-DSP-mix cells were transfected with the spike, either alone or in combination with its receptor ACE2, and cell–cell fusions quantified through measurement of fluorescence (Figure 2a) as well as bioluminescence signals (Figure 2b). Intriguingly, we found that the SARS-CoV-2 spike protein showed a considerable fusion activity even in the absence of ACE2, although the fusion activity was significantly enhanced upon co-expression of the receptor (Figure 2a,b). This effect was specific for the spike of SARS-CoV-2 as the spike protein of SARS-CoV-1 was fusion-inactive in the DSP assay and comparable to the hemagglutinin glycoprotein of avian influenza virus strain H7N7 (H7N7 HA), HCMV gB or the human immunodeficiency virus type 1 (HIV-1) envelope glycoprotein gp160 (Figure 2a,b). Moreover, even following co-expression of its cognate receptor ACE2, the SARS-CoV-1 spike remained fusion-negative and could only be activated upon co-expression of ACE2 and the transmembrane serine protease TMPRSS2. These findings clearly demonstrated the different fusion characteristics of both coronavirus spike proteins. Of note, the SARS-CoV-2 spike showed a similar degree of fusogenicity in the absence of ACE2 to the viral fusion protein, VSV-G (Figure 2b), which is well described in terms of its broad cellular tropism and its common usage for viral pseudotyping [32]. As determined through confocal microscopy (Figure 2c), in the absence of ACE2, only a few of the cells expressing the SARS-CoV-2 spike induced the fusion, which consisted predominantly of two neighboring cells (Figure 2c, panels a–d), while large multinucleated syncytia were detectable following ACE2 receptor overexpression (Figure 2c, panels e–h). To exclude that the low level of expression of endogenous ACE2 was responsible for the spike-induced fusions seen in the 293T-DSP-mix cells (Figure 2a,b), an entry assay using spike pseudotyped lentiviral particles was performed (Figure 2d). As evident from Figure 2d, only upon ectopic expression of ACE2 could viral particles enter the cell and luciferase reporter activity be measured (Figure 2d). This is in accordance with observations from our and other groups that HEK293T cells cannot readily be infected with SARS-CoV-2 virus ( [33]. Collectively, these data demonstrate that in contrast to the entry process of SARS-CoV-2, which critically depends on ACE2 and yields in large syncytia, the SARS-CoV-2 spike rarely induces ACE2 receptor-independent cell–cell fusions, which typically do not exceed the number of two neighboring cells. 

### 3.3. Proteolytic Cleavage of Spike by Furin but Not TMPRSS2 Is Essential for ACE2-Independent Cell–Cell Fusions

**Figure 3:**
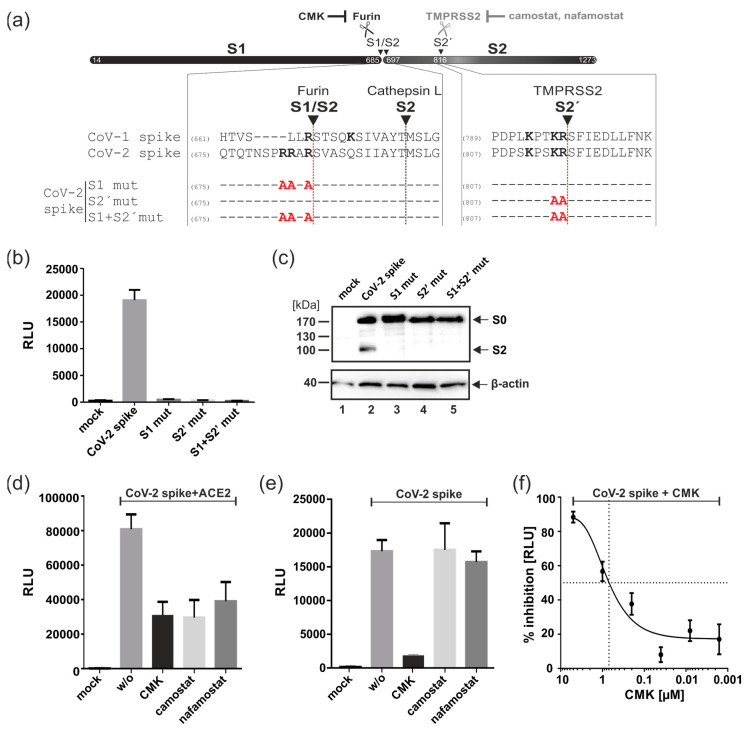
Proteolytic cleavage of the SARS-CoV-2 spike protein by furin but not TMPRSS2 is required for ACE2-indpendent cell–cell fusions. (**a**) Top: Schematic of SARS-CoV-2 spike, which is proteolytically processed by the cellular proteases furin, Cathepsin L and/or TMPRSS2 into its S1, S2 and S2′ subunits, and this cleavage can be blocked by CMK or camostad/nafamostat, as indicated. The magnification shows an amino acid alignment of the respective protease cleavage sites of spike proteins encoded by SARS-CoV-1 [YP_009825051.1] and SARS-CoV-2 [YP_009724390.1]. Numbers indicate the position of the bordering amino acids; basic residues potentially involved in recognition by cellular proteases are highlighted in bold font. Mutants that harbor alanine substitutions in the protease cleavage sites of SARS-CoV-2 spike are indicated at the bottom. (**b**) DSP fusion assay using 293T-DSP-mix cells transfected with plasmids encoding CoV-2 spike or the respective proteolytic cleavage site mutants as indicated. (**c**) Western blot analysis using lysates of HEK293T cells transfected with plasmids encoding the indicated spike proteins. Beta-actin detection served as a loading control. (**d**–**f**) DSP fusion assay with 293T-DSP-mix cells co-expressing ACE2 and CoV-2 spike (**d**) or expressing CoV-2 spike alone (**e**,**f**) were treated at 4h post-transfection with the furin inhibitor CMK (10 µM), serial dilutions thereof (**f**) or the TMPRSS2 protease inhibitors camostat (100 µM) and nafamostat (20 µM), respectively (**d**,**e**). Nontreated cells (*w*/*o*) served as internal controls. Cell–cell fusions were quantified through bioluminescence detection following addition of the substrate EnduRen. (**f**) The data from (**e**) were used to calculate the CMK-mediated inhibition of fusion in percent based on the RLU in relation to the nontreated control.

The DSP fusion assay revealed substantial differences in the fusion activity of the SARS-CoV-1 and SARS-CoV-2 spike proteins. As illustrated in Figure 3a, the SARS-CoV-2 spike (CoV-2 spike) has evolved a multibasic cleavage site (RRAR) at the S1/S2 boundary, which has been shown to confer functional advantages like enhanced cell–cell fusion and syncytium formation compared to the spike of SARS-CoV-1 (CoV-1 spike) [7,34]. In order to induce membrane fusion, the SARS-CoV-2 spike must be cleaved at the S1/S2 and the S2′ site by host cell proteases like furin and TMPRSS2 (Figure 3a). In accordance with this mechanism, mutation of either the S1/S2 (S1mut; mutation of R682, R683 and R685 to A) or S2′ (S2′mut; mutation of K814 and R815 to A) cleavage site alone or in combination (S1+S2′mut) resulted in a complete loss of the spike’s fusion activity following transfection of the 293T-DSP-mix cells (Figure 3b). Western blot analysis confirmed the efficient expression of the respective mutants and their defect in proteolytic processing as indicated by the missing S2 band (Figure 3c). Having also shown that for ACE2 receptor-independent cell–cell fusion, the spike protein has to be processed at both proteolytic cleavage sites (S1/S2 and S2′), we next investigated which cellular proteases are involved in this process. Generally, it is thought that the spike protein is initially cleaved by furin at the S1/S2 boundary during its biosynthesis [15]. This likely promotes the binding of the spike to the ACE2 receptor, thereby priming it for TMPRSS2 cleavage at the S2′ site, leading to exposure of the FP [15]. To block the proteolytic processing of the spike, the furin inhibitor CMK or the TMPRSS2 inhibitors camostat and nafamostat were added to the transfected 293T-DSP-mix cells overexpressing either the spike protein together with ACE2 (Figure 3d) or the spike protein alone (Figure 3e,f). As expected, in the presence of ACE2, the inhibition of furin or TMPRSS2 significantly reduced the spike’s fusion activity, confirming the activity of the inhibitors (Figure 3d). Importantly, in the absence of ACE2, only the furin inhibitor CMK prevented spike-induced membrane fusions, while treatment with the TMPRSS2 inhibitors camostat and nafamostat was completely ineffective (Figure 3e). The latter observation further strengthens the conclusion that the spike protein alone is capable of inducing cell fusions in an ACE2 receptor-independent manner in HEK293T cells. Nonetheless, since both proteolytic cleavage sites of the spike (S1/S2 and S2′) were shown to be of importance (Figure 3b), this finding raises the possibility that furin was capable of also cleaving the spike protein at S2′ without a requirement for ACE2 receptor engagement. Lastly, the half-maximal inhibitory concentration (IC_50_) for CMK was determined through DSP assay as 0.7 μM (Figure 3f) and the 50% cytotoxic concentration (CC_50_) of CMK was 620.2 μM, yielding a selectivity index (SI = CC_50_/IC_50_) of 886. Therefore, pharmacological inhibition of furin cleavage represents a feasible approach to interfere with the spike’s protein receptor-independent fusion activity, and this suggests that the DSP assay could be a valuable tool for the screening and identification of such inhibitors.

### 3.4. Spike Variants Greatly Differ in Their Capacity to Induce Cell Membrane Fusions

**Figure 4:**
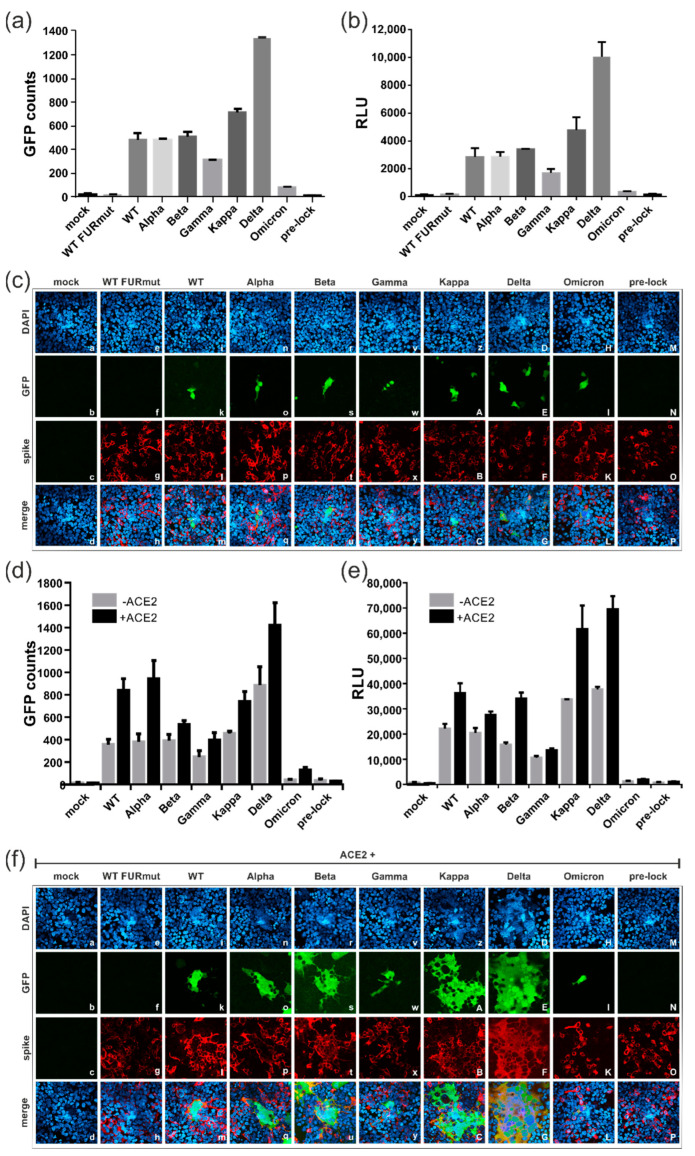
Spike proteins from SARS-CoV-2 variants of concern greatly differ in their capacity to induce cell–cell fusions. (**a**–**f**) 293T-DSP-mix cells were transfected with empty vector (mock) or plasmids encoding CoV-2 spike wildtype (WT), mutants (FURmut and pre-lock) or the indicated VOC either alone (**a**–**e**) or upon co-transfection with a plasmid coding for ACE2 (**d**–**f**). Cell–cell fusions were quantified using the DSP assay for GFP counts as determined with the CTL-fluorospot reader (**a**,**d**) or luciferase reporter activity (**b**,**e**). (**c**,**f**) Immunofluorescence analyses of cell–cell fusions in the absence (**c**) or presence (**f**) of exogenous ACE2 expression. GFP signals of the reconstituted DSP reporter proteins were imaged using confocal laser scanning microscopy. The spike variants were stained using the human anti-spike antibody 4C12 and cell nuclei were visualized using DAPI staining.

Since the outbreak in 2019, multiple SARS-CoV-2 variants of concern (VOCs) have emerged. To compare the fusion capacity of the spike protein from the strain Wuhan-Hu-1 (WT) with spike variants derived from Alpha, Beta, Gamma, Kappa, Delta and Omicron, expression constructs were transfected into 293T-DSP-mix cells (Figure 4). As evident from Figure 4a,b and in contrast to negative controls like the furin cleavage site spike mutant S1mut (see Figure 3a), hereafter referred to as WT FURmut, or a mutant that is locked in the prefusion state (pre-lock), all variants could induce cell–cell fusions in the absence of ACE2. However, the individual spike protein derivatives differed significantly in their fusion activity. The Delta spike protein demonstrated the highest fusion activity when compared to the other variants (Figure 4a,b). In contrast to that of WT, Alpha, Beta, Gamma and Kappa showed an intermediate phenotype, and the Omicron spike protein exhibited little cell fusion activity (Figure 4a,b). As determined through confocal microscopy (Figure 4c), the enhanced fusogenicity of the Delta spike did not result from the formation of large syncytia, as only two-cell fusions were detectable when ACE2 was absent (Figure 4c, panel E). Rather, Delta spike expression resulted in more GFP-positive cells per image compared to the other variants (Figure 4c, compare panel E with panels f, k, o, s, w, A, I and N). Again, for all variants, the cell–cell fusion activity could be significantly enhanced by co-transfection of ACE2 (Figure 4d,e), which led to the formation of large multinucleated cell syncytia (Figure 4f). The size of the respective syncytia correlated with the degree of fusion activity of the single spike variants, with Omicron only showing very small fusions, even in the presence of ACE2 (Figure 4f, panels f, k, o, s, w, A, E, I, and N). Thus, the individual spike variants significantly differ in their fusogenicity irrespective of whether ACE2 is present or not, ranging from extremely fusogenic (Delta) to almost fusion-negative (Omicron).

### 3.5. All Spike Variants Are Dependent on Furin Cleavage for Induction of ACE2-Independent Cell Fusions

**Figure 5:**
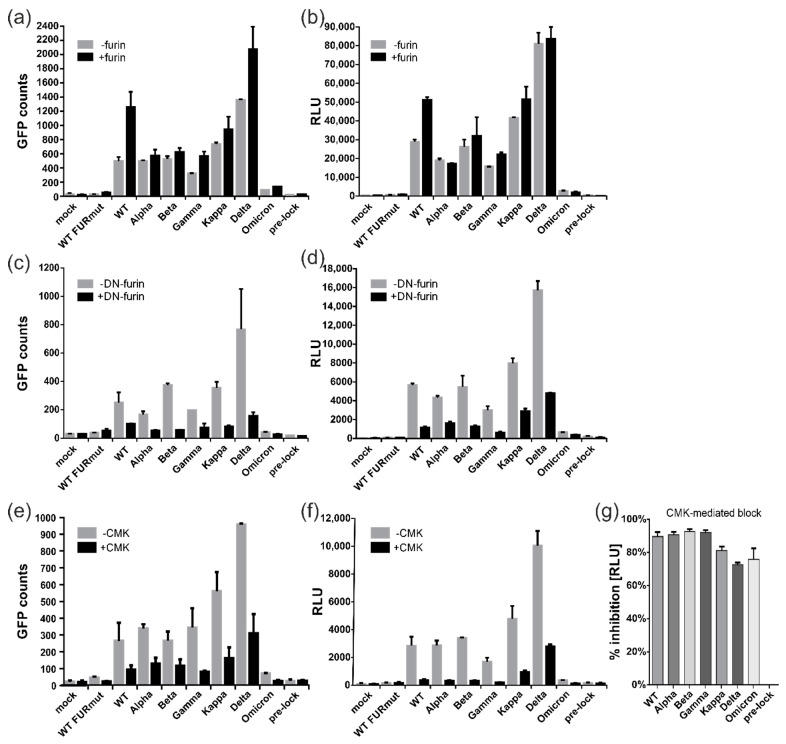
All tested SARS-CoV-2 spike proteins from variants of concern are dependent on furin cleavage for induction of ACE2-independent cell–cell fusions. (**a**–**g**) DSP assay with 293T-DSP-mix cells transfected with empty vector (mock), CoV-2 spike wildtype (WT), mutants thereof or variants of concern as indicated. (**a**–**d**) The spike-encoding plasmids were co-transfected together with wildtype furin (**a**,**b**, +furin) or its catalytically inactive derivative D153N, thus serving as a dominant negative mutant termed DN-furin (**c**,**d**, +DN-furin). (**e**,**f**) For pharmacological inhibition of endogenous furin, the transfection mix was replaced at four hours post-transfection with medium containing 10 µM of the furin inhibitor CMK (+CMK). Cell–cell fusions were quantified via GFP (**a**,**c**,**e**) or luciferase activity (**b**,**d**,**f**). (**g**) The data obtained in (**f**) were used to calculate the CMK-mediated inhibition of fusion in percent based on the RLU in relation to the respective variant not treated with the inhibitor.

Having shown that spike proteins from all variants could induce cell–cell fusions without receptor engagement (see Figure 4a,b), we addressed the question of whether this fusion activity was dependent on proteolytic cleavage by furin, as demonstrated for the WT spike protein (see Figure 3). Initially, the spike proteins from individual variants were expressed either alone or in combination with furin (Figure 5a,b). However, with the exception of the WT spike, overexpression of the proprotein convertase led to only a slight enhancement of the fusion activity of the spike proteins from other variants (Figure 5a,b). To rule out that the spike proteins were being cleaved by the endogenous furin, an enzymatically inactive form of furin that functioned as a dominant negative mutant (DN-furin) was co-transfected (Figure 5c,d). As predicted, overexpression of the DN-furin resulted in a decrease in ACE2-independent fusion activity of the spike proteins of all the variants (Figure 5c,d). A similar reduction in the fusion activity could be achieved through treatment of the transfected cells with the furin protease inhibitor CMK (Figure 5e,f). Of note, the inhibitory effect of CMK was comparable for all variants, ranging from 70 to 95% of fusion inhibition (Figure 5g). This result indicated that the residual amount of fusion activity that was detectable in the case of the Omicron spike was likewise dependent on efficient furin cleavage. Hence, for all SARS-CoV-2 variants, cleavage of the spike by the proprotein convertase furin played a pivotal role in activating the fusion process without receptor engagement, irrespective of whether it was a highly fusogenic spike protein from a VOC or not.

### 3.6. Characterization of the ACE2-Independent Fusion Activity of Spike

**Figure 6:**
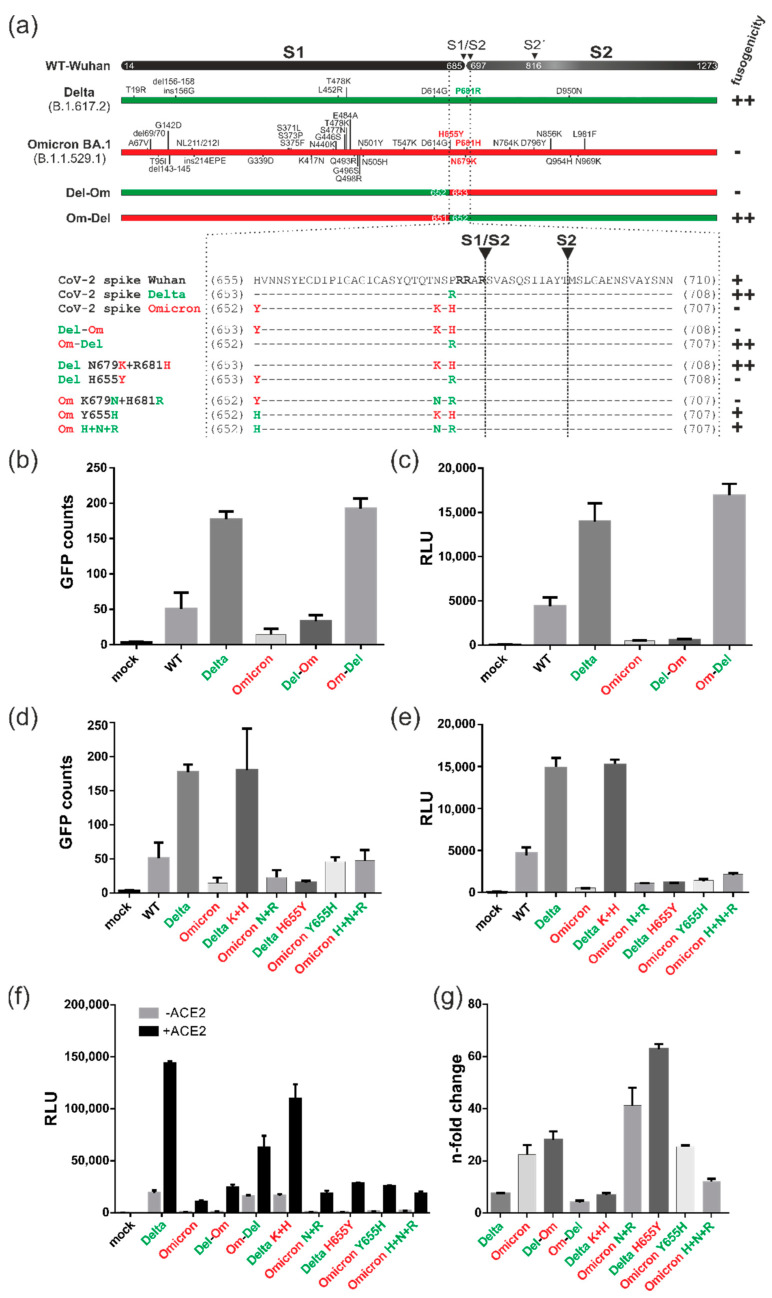
Characterization of SARS-CoV-2 spike’s fusion activity in the absence of ACE2. (**a**) Schematic of SARS-CoV-2 spike wildtype (WT, Wuhan-Hu-1) and the VOCs Delta and Omicron, as well as chimeric proteins and point mutants thereof. Upper part: Acquired mutations in Delta and Omicron spike are indicated. Lower part: The magnification depicts the corresponding amino acid sequences in proximity to the respective protease cleavage sites. The number in the name of the mutant refers to the amino acid position in WT spike. (**b**–**f**) DSP assays of 293T-DSP-mix cells expressing the respective spike variant, chimera or mutant as indicated. Cell–cell fusions were quantified via GFP using the CTL-fluorospot reader (**b**,**d**) or bioluminescence upon Renilla-luciferase mediated cleavage of EnduRen (**c**,**e**,**f**). (**f**) Fusion activity of the individual spike protein in the presence (+ACE2) or absence of ACE2 (-ACE2) as measured by luciferase activity. (**g**) Data from (**f**) were used to calculate the n-fold change in fusion activity of the respective spike derivative following ACE2 co-expression relative to their fusion activity when expressed alone.

To dissect the determinants of the fusion activity of the spike protein in the absence of ACE2 in further detail, we compared the protein sequence of the highly fusogenic Delta variant (B.1.617.2) with that of the almost non-fusogenic Omicron subtype BA.1 (B.1.1.529.1). As depicted in the sequence alignment of Figure 6a, the Omicron spike acquired a remarkably high number of novel mutations, which were distributed throughout the entire protein. Initially, to determine whether the fusion activity was predominantly regulated by the N- or the C-terminal domain of the spike protein, the chimeric proteins Del-Om (aa 1-652 of the Delta spike and aa 653-1273 of the Omicron spike) and Om-Del (aa 1-651 of Omicron and aa 652-1273 of Delta) were generated (Figure 6a). The expression of these chimera in the 293T-DSP-mix cells revealed that Del-Om behaved exactly like Omicron, while Om-Del showed a comparably high fusion activity similar to that of the spike protein from the Delta variant (Figure 6b,c). These data led to the conclusion that the fusogenic potential of the spike protein is almost entirely dependent on the C-terminal domain of the Delta spike protein comprising aa 652-1273 (see Figure 6a). Of note, this region includes both proteolytic cleavage sites S1/S2 and S2′, which are of major relevance for the fusogenicity of the spike (see Figure 3b). Importantly, the Omicron spike protein acquired three novel key mutations (H655Y, N679K and P681H) near or directly at the furin cleavage site (see Figure 6a). Although these mutations are thought to enhance proteolytic cleavage, experimental evidence revealed that relatively, the Omicron spike protein is poorly cleaved, especially when compared to the Delta spike protein [35]. To directly explore the role of these mutations in the fusion activity of the spike protein, we exchanged the aa 679 and 681 directly located at the S1/S2 cleavage site in the Omicron and Delta spike with their respective counterparts, giving rise to Delta N679K+R681H (Delta K+H) and Omicron K679N+H681R (Omicron N+R) (see Figure 6a). Surprisingly, exchange of the indicated aa did not impact the cell–cell fusion activity of either exchange mutants as both mutants showed the same degree of fusion activity as their respective WT counterparts (Figure 6d,e; compare Delta K+H with Delta and Omicron N+R with Omicron). Next, we carried out an aa exchange at position 655 and generated the Delta H655Y and Omicron Y655H mutants, respectively (see Figure 6a). 

Intriguingly, the introduction of the H655Y mutation completely abolished the cell–cell fusion properties of the Delta spike (Figure 6d,e; compare Delta H655Y to Delta). The Omicron exchange mutant Y655H, in contrast, showed enhanced fusion activity that was nearly three times greater than the wildtype Omicron spike protein (Figure 6d,e; compare Omicron Y655H to Omicron). Although Omicron Y655H did not reach the fusion levels of the Delta spike, its fusion activity was similar to the WT (Wuhan-Hu-1) spike (Figure 6d,e; compare Omicron Y655H to Delta and WT). To exclude that the introduced amino acid substitutions affected the structural integrity of the spike mutants, the DSP fusion assay was repeated in the presence of ACE2 (Figure 6f,g). As shown in Figure 6f, for all chimeras and mutants, a considerable amount of fusion activity was detectable following ACE2 receptor overexpression. Notably, mutants such as Delta H665Y with low fusogenic potential in the absence of ACE2 showed the greatest increase in fusion activity following ACE2 engagement (Figure 6g). These results demonstrated that all of the spike protein mutants and chimeras generated for this series of experiments were functional. Thus, it can be concluded from these mapping studies that aa 679 and 681, which are in close proximity to the furin cleavage site, are not involved in regulating the receptor-independent membrane fusion activity of the spike protein, while aa 655, located further upstream of the S1/S2 cleavage site, seems to have a significant role in this fusion process.

### 3.7. Blocking of Spike-Induced Cell Fusions by Antibodies

**Figure 7:**
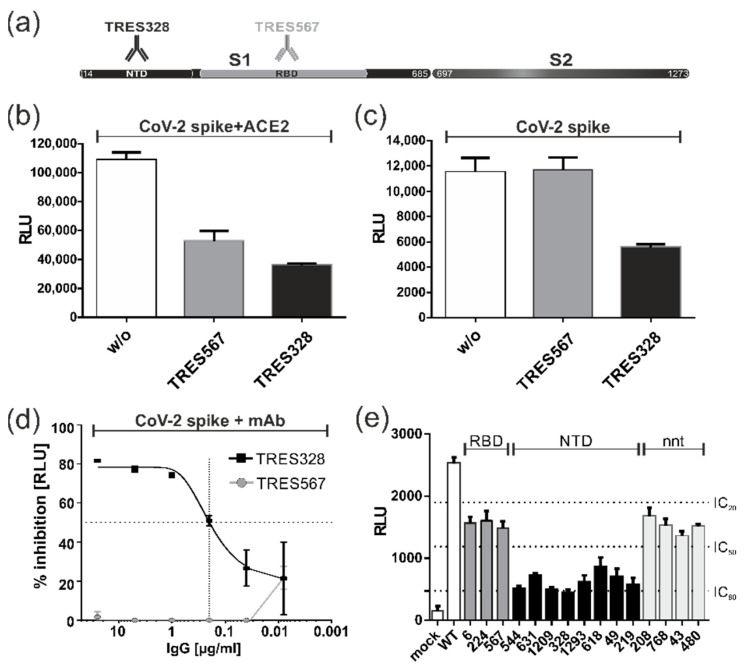
Blocking SARS-CoV-2 spike’s fusion activity with monoclonal antibodies. (**a**) Schematic representation of SARS-CoV-2 spike with its S1 and S2 subunits and the target sites of the spike-specific monoclonal antibodies TRES328 (NTD) and TRES567 (RBD). (**b**–**e**) DSP assays with 293T-DSP-mix cells transfected with spike in combination with ACE2 (**b**) or spike alone (**c**–**e**). At 4h post-transfection, 25 µg/mL (**b**,**c**,**e**) or serial dilutions (**d**) of the indicated neutralizing anti-spike antibodies directed against its NTD or RBD were added, and non-neutralizing (nnt) anti-spike antibodies served as internal controls (**e**). Cell–cell fusions were quantified through bioluminescence detection following addition of the substrate EnduRen. (**d**) The obtained data were used to calculate the antibody-mediated inhibition of fusion in percent based on the RLU in relation to the nontreated control.

Finally, we addressed the question of whether the fusion activity of the spike as measured in the DSP cell fusion assay could be blocked by antibodies directed at the spike protein. The N-terminal domain (NTD) and the receptor-binding domain (RBD) of the spike have been identified as critical targets for the development of effective therapeutic antibodies against SARS-CoV-2 [36]. Thus, in an initial approach, two spike-specific mAbs (TRES328 and TRES567) targeting the respective structural domains were tested. The antibodies were isolated from TRIANNI mice immunized with WT Wuhan-Hu-1 and have been shown to neutralize infection in the subnanomolar range, thereby protecting from disease, in a SARS-CoV-2 infection model [30]. As indicated in Figure 7a, TRES328 has been mapped to bind to the N-terminal domain (NTD) of the spike, while TRES567 was shown to target the receptor-binding domain (RBD) and to compete with ACE2 binding [28,30]. When added to the DSP fusion assay at 4h after transfection of the cells, both mAbs significantly reduced the fusion activity of the spike protein from WT Wuhan-Hu-1 in the presence of ACE2 (Figure 7b). However, in the absence of ACE2, only the NTD mAb TRES328 prevented receptor-independent cell fusions of the spike, as the RBD mAb TRES567 was nearly inactive under these conditions (Figure 7c). Serial dilutions of the antibodies revealed an IC_50_ of TRE328 (NTD) in the nanomolar range (~0.2 µg/mL), while TRE567 (RBD) had no inhibitory effect at any of the concentrations tested in the absence of ACE2 (Figure 7d). In agreement with their binding profiles, these observations further strengthen the argument that the spike protein can induce cell fusions in an ACE2 receptor-independent manner. This conclusion was further supported by the including a number of additional NTD- and RBD-binding mAbs in the DSP fusion assay (Figure 7e). Again, only antibodies binding to the NTD of the spike (TRES544, TRES631, TRES1209, TRES328, TRES1293, TRES618, TRES49 and TRES219) were able to block cell fusions without receptor engagement, while the RBD mAbs TRES6, TRES224, and TRES567 were as ineffective as several non-neutralizing (nnt) anti-spike mAbs (TRES208, TRES768, TRES43, TRES480), which were included as negative controls. Taken together, these results clearly demonstrate that among the mAbs assayed in these studies, only NTD-directed antibodies were capable of blocking the ACE2-independent membrane fusion induced by the spike protein, whereas antibodies that have been shown to prevent the entry process of SARS-CoV-2 by negatively affecting the interaction of the spike protein with its cognate receptor ACE2 did not block receptor-independent cell–cell fusion. 

## 4. Discussion

The COVID-19 pandemic caused by infection with SARS-CoV-2 represented an unprecedented public health crisis that also resulted in a considerable economic burden in all regions of the world. The molecular mechanisms that contribute to the dissemination of the virus and induction of tissue damage and disease within its host are still not entirely understood. It is generally accepted that in the presence of its receptor ACE2, the SARS-CoV-2 spike protein induces cell–cell fusion, leading to large multinucleated syncytia, which provide an additional means for dissemination in vivo and contribute to the pathogenesis of SARS-CoV-2 infections. In fact, histopathological findings in autopsy reports from COVID-19 patients and non-human primate models have consistently included the presence of infected multinucleated syncytial lung tissues [21,37,38,39]. One mechanistic explanation for this observation is based on the SARS-CoV-2 entry process that relies on the membrane protease TMPRSS2, which upon binding of the SARS-CoV-2 spike protein to the receptor ACE2, faciltates membrane fusion [12]. This entry-like syncytia formation can be recapitulated in vitro upon infection of ACE2-positive Vero, CaCo-3 or Calu-2 cells with SARS-CoV-2 as well as upon ectopic expression of SARS-CoV-2 spike protein in these cell lines [19,23,30].

We established a highly sensitive membrane fusion assay that allowed the unbiased quantification of split GFP and luciferase reporter protein complementation upon expression of a viral fusion protein in combination with its receptor (Figure 1a). This DSP assay may serve as a surrogate entry assay for SARS-CoV-2, as we documented large multinucleated syncytia when the SARS-CoV-2 spike protein was co-expressed with ACE2, leading to elevated GFP-fluorescence and luciferase activity (Figure 2). Importantly, in contrast to infection experiments using highly pathogenic SARS-CoV-2, the DSP assay can be performed under biosafety level 1 (BSL-1) containment to characterize spike protein fusion activity and to screen for antibodies or compounds that inhibit the fusion process (Chen and Reuter et al., unpublished). Unexpectedly, the highly sensitive DSP assay revealed that the SARS-CoV-2 spike protein-induced syncytia formation was independent of expression of the ACE2 receptor. This conclusion was based on results from several controlled experiments. First, 293T-DSP-mix cells fused following SARS-CoV-2 spike protein expression, even though these cells expressed insufficient amounts of ACE2 for infection with lentiviral particles pseudotyped with spike protein (Figure 2d) or authentic cell-free SARS-CoV-2 (own observation and [33]). Second, spike proteins encoded by SARS-CoV-1 or by the alphacoronavirus HCoV- NL63, which also use ACE2 as an entry receptor, did not induce membrane fusion in the DSP assay (see Figure 2 and [40,41]. Third, mAb TRES567, which binds to the RBD of the SARS-CoV-2 spike and has been shown to block ACE2-dependent syncytia formation, failed to block membrane fusion in HEK293T-DSP-mix cells that expressed the spike protein alone (Figure 7). Lastly, the pharmacological interference of the spike protein RBD interaction with ACE2 with the TMPRSS2 inhibitors camostat/nafamostat (Figure 3e) or TMPRSS2 overexpression had no impact on SARS-CoV-2 spike protein-induced syncytia formation in the absence of ACE2 expression. 

Of note, ACE2-independent membrane fusions were rarely observed in a small proportion of SARS-CoV-2 spike expressing cells, as illustrtated by the amount of GFP-fluorescence compared to the number of cells stained with an anti-spike protein mAb (Figure 2c, compare panels c and b or Figure 4c,f). The fact that HEK293T cells cannot be infected with cell-free virus makes the utilization of any of the other proposed auxiliary entry factors like the C-type lectins DC-SIGN and L-SIGN [42], receptors like Nrp1 [43,44] B0AT1 [45], KREMEN1 and ASGR1 [46] or IFITM2 [47] unlikely. These data raise the possibility that yet unidentified cellular factors can induce or repress the fusion activity of the spike protein, possibily via cellular signaling or regulatory cellular membrane proteins (e.g., IFITMs and Ly6E) [48,49].

As described above, the fusogenic activity of the spike protein requires activation through proteolytic cleavage carried out by the cellular proteases furin and TMPRSS2 at its S1/S2 and S2′ site, respectively. In accordance with the requirement for cleavage of the spike protein in this mechanism, our mapping studies revealed that these two cleavage sites were critically important for receptor-independent membrane fusion (Figure 3b,c). However, in agreement with the ACE2-independent fusion activity of the spike protein, we found that neither overexpression nor pharmacological inhibition of TMPRSS2 with the established inhibitors camostat and nafamostat had any effect on cell–cell fusion in the absence of ACE2 (Figure 3). In contrast, the cleavage of the spike protein by furin was critical, since overexpression of catalytically active furin enhanced cell fusion activity, whereas DN-furin or pharmacological inhibition using CMK decreased cell fusion activity in the absence of ACE2 (Figure 3 and Figure 5). Intriguingly, none of the viral fusogens that are also proteolytically processed by furin, i.e., human immunodeficiency virus Env [50], human cytomegalovirus gB [51,52], the H7 subtype HA of the avian influenza virus [53,54] or the SARS-CoV-1 spike, which is cleaved by TMPRSS2 [55], induced cell–cell fusions in the absence of ACE2 (Figure 2a,b). These viral fusogens share a common synthetic pathway in that they are proteolytically cleaved only once during their biosynthesis, a pathway which is in contrast to the double-cleaved spike of SARS-CoV-2. Consistent with this hypothesis were findings reported as this manuscript was being prepared that demonstrated that furin, in addtion to its cleavage of the S1/S2 junction, also cleaves the spike protein at its dibasic residues lysine and arginine at positions 814 and 815 within the S2′ site (Figure 3a). This site may also serve as a substrate for TMRPSS2 [56,57,58]. However, in contrast to these findings obtained in ACE2-positive cells, our study clearly demonstrated SARS-CoV-2 spike protein-induced membrane fusion in cells that are devoid of this receptor. In this context, it is important to note that although furin predominantly accumulates and functions in the trans-Golgi network (TGN), it can be further transported to the cell surface and back via the endosomal pathway and is also shed into the extracellular space upon proteolytic separation from its membrane anchor [59,60]. The complementarity and interchangeability of the proteases TMPRSS2 and furin raises the possibility that in the absence of ACE2 and/or TMPRSS2, membrane-anchored furin provides a signal that senses the proximity between an infected cell and its target cell, which then, in turn, triggers fusion through the proteolytical cleavage of the spike protein in trans. In this case, furin might be considered as an additional entry receptor for fusion proteins like the SARS-CoV-2 spike that are efficiently cleaved and preactivated, e.g., by furin within the TGN before they are transported to the surface, where an additional cleavage is mediated via TMPRSS2 or furin in trans to initiate the fusion process. In contrast, the CoV-1 spike and related proteins that are insufficiently cleaved within the TGN are more dependent on ACE2-binding, followed by cleavage via TMPRSS2 at the monobasic S1 site or via TMPRSS2 and furin at the dibasic S2′ site (Figure 3a). The capacity of SARS-CoV-2 to spread from infected ACE2-positive cells to neigboring ACE2-negative cells has also been reported by other groups upon”Inf’ction with spike pseudotyped lentiviral particles or authentic virus, and this might have important implications in its pathogenicity [18,61]. Our study provides a mechanistic explanation for these observations. Thus, it is tempting to speculate that the ACE2-independent cell–cell spread not only extends the tropism of SARS-CoV-2 and its intra-host dissemination but further affects its pathogenicity, i.e., via immune cell elimination or evasion from antibody-mediated neutralization of cell-free virus [20].

By comparing the spike proteins encoded by previous and current variants of concern (VOCs), we found substantial differences in their receptor-dependent as well as receptor-independent fusion activity. The Delta spike was extremely potent in inducing membrane fusions compared to the Alpha, Beta, Gamma and Kappa variants. This finding can be explained by the more basic S1/S2 cleavage in Delta, which likely enhances its preactivation within the TGN (compare Figure 3a and Figure 6a). In contrast, the Omicron spike protein almost completely lacked fusion activity. This finding seems rather surprising as N679K in Omicron resulted in an additional basic residue next to its polybasic sequence, which, therefore, should increase its furin cleavage and membrane fusion activity as compared to the Delta spike. In contrast, our mapping analyses revealed that aa 655–1273 and particularly H655Y were of greater importance, while aa directly at the furin cleavage site were not of major relevance for the ACE2-independent fusion activity of the spike protein (Figure 6). In agreement with our results, the SARS-CoV-2 Omicron mutation H655Y was recently reported as a critical regulator of the entry properties of the spike protein, leading to the preferential usage of endosomal entry over the cell-surface membrane fusion pathway ([62] and Yamamoto et al. 2022, bioRxiv preprint server: doi: https://doi.org/10.1101/2022.03.21.485084 [63]). However, these studies were carreid out in ACE2-positive cells, hence recapituling the entry process. In contrast, our study addressed the spike protein impact on the cell that expressed this protein and clearly documented that residue 655 is also critical for receptor-independent cell–cell fusion. Importantly, the Gamma variant also encodes the same H655Y substitution, but neither is it known to demonstrate the entry preference of Omicron nor did it fuse less efficiently (Figure 4 and Figure 5). These findings strongly suggest that additional amino acid substitutions of the Omicron spike contribute to the mechanism leading to receptor-independent fusion that is described in this manuscript. Taken together, our study revealed that the amino acid composition of the S1/S2 and S2′ cleavage sites themselves were neither predictive of the susceptibility to cleavage by furin nor the receptor-independent fusion acitivity of the spike. This is of particular importance since SARS-CoV-2 has the capacity to evolve within its host [64] and recombine [65], e.g., with novel animal-adapted lineages [66], which may have unpredictable consequences on the pathogenicity of the emerging strain as well as on the efficacy of COVID-19 vaccines. Among the currently approved vaccines requiring de novo expression in the host are some that encode a diproline mutated spike (pre-lock), as for instance BNT162b2 (Pfizer, BioNTech) or mRNA-1273 (Moderna) [67,68]. Such a prefusion stabilized spike protein did not induce cell–cell fusions in our DSP assays, irrespective of whether the ACE2 receptor was present or absent (Figure 4 and Figure 5). One might speculate that other vaccines like AZD1222 (AstraZeneca), encoding the full-length codon-optimized wildtype spike protein [69], are more prone to adverse side-effects as a result of their receptor-dependent and -independent fusogenicity (Figure 2 and Figure 5) and which, in fact, have been observed in vaccinees [70,71,72,73,74]. We propose that the use of the DSP assay is to analyze novel VOCs, recombinants and/or vaccine candidate spike proteins in order to quantify their membrane fusion activity as well as their potential escape from the antiviral countermeasures.

Cell–cell fusion, dependent or independent of the ACE2 receptor, is of major importance for the dissemeniation and pathogenicity of SARS-CoV-2 and therefore represents an attractive target for pharmacological intervention. Since we have identified the critical role of furin cleavage at the S1/S2 and S2′ sites as important determinants of SARS-CoV-2’s receptor-independent cell-to-cell transmissability, we attempted to block this fusion activity with the furin inhibitor CMK (Figure 3 and Figure 5). Notably, the IC_50_ of CMK in our assay was approximately 12 times higher than the inhibitory concentration required for suppressing virus production and cytopathic effect in ACE2 receptor-positive Vero-E6 cells [17]. This can be explained by the continuous supply of novel proteins from the transiently transfected HEK293T cells in our in vitro DSP assay. However, since the inhibitory concentration was almost 900-fold lower than the CC_50_, it is feasible to consider a strategy of furin inhibition, possibly in combination with TMPRSS-inhibtors, as a therapeutic option for COVID-19 patients (reviewed in [75,76]).

In an alternative blocking approach, our studies revealed that anti-spike protein mAbs prevented spike-induced cell–cell fusions (Figure 7a–c). In accordance with our finding of ACE2-independent membrane fusion, mAb TRES567, which is directed against the RBD of the spike, failed to block fusion. In contrast, TRES328, which targets the NTD, blocked cell–cell fusion [30]. Similar observations were obtained for mAb 7D10, which is directed against the NTD of MERS-CoV spike and prevents the prefusion to post-fusion transition of the S2 subunit and therefore the initiation of membrane fusion [77]. It is tempting to suggest that blocking the fusion function of the spike could be one mechanism of TRES328-mediated virus neutralization, as well as its interference with spike protein-induced cell–cell fusion. Likewise, our group has shown that syncytium formation of an intrinsically fusogenic human CMV gB/VSV-G chimera can be significantly inhibited by only a subset of neutralizing mAbs that target a structural domain of gB containing its fusion loops [27]. In both experimental systems, the experimentally derived IC_50_ of the anti-gB or anti-spike protein antibodies for fusion inhibtion was about 10–20-fold higher than their IC_50_ neutralizing activity, which can be explained by the continous supply of newly synthesized surface glycoproteins in our in vitro fusion assays [27,28,30]. Nevertheless, the DSP cell fusion assay represents a valuable tool for the identification of fusion-inhibiting antibodies that are directed against the functionally important and more conserved S2 subunit of the SARS-CoV-2 spike. In fact, peptide scanning analyses in COVID-19 convalescent sera revealed dominant antibody responses against the S2 subunit, containing the fusion peptides and heptad repeats HR1 and HR2 [78,79]. These results not only illustrate the accessability of functionally important domains during the course of infection but also for pharmacological intervention with therapeutic mAbs or inhibitors directed against the S2 subunit. The highly sensitive DSP assay allowed us to identify a thus far poorly described mechanism of ACE2 receptor-independent cell–cell fusion, and the detailled characterization of the mechanism behind this process will provide the basis for future studies that will enable the identification of potential new and more effective therapies than are currently available.

## Figures and Tables

**Table 1:** Primers used in this study for cloning or site-directed mutagenesis.

Primer No.	Name	Sequence
2-58	5AscI,H3,Kozakhu+muACE2	GCATGGCGCGCCAAGCTTGCCACCATGTCMAGCTCYTCCTGGCTCCTTC
2-59	3Pme1,NotSTOPhu+muACE2	GCATGTTTAAACGCGGCCGCCTAAAAGGARGTCTGARCATCATC
3-71	cCoV-2 spike mutS1	CCAGACACAGACAAACAGCCCCGCAGCGGCCGCATCTGTGGCCAGCCAGAGCATC
3-72	nCoV-2 spike mutS1	GATGCTCTGGCTGGCCACAGATGCGGCCGCTGCGGGGCTGTTTGTCTGTGTCTGG
3-73	cCoV-2 spike mutS2′-AA	CGATCCTAGCAAGCCCAGCGCGGCGAGCTTCATCGAGGACCTGC
3-74	nCoV-2 spike mutS2′-AA	GCAGGTCCTCGATGAAGCTCGCCGCGCTGGGCTTGCTAGGATCG
3-91	5mutSpike-K986P V987P	CGATATCCTGAGCAGACTGGACCCGCCGGAAGCCGAGGTGCAGATCGAC
3-92	3mutSpike-K986P V987P	GTCGATCTGCACCTCGGCTTCCGGCGGGTCCAGTCTGCTCAGGATATCG
4-11	n dominant negative Furin	GCATTGTGGTCTCCATTCTGAACGATGGCATCGAG
4-12	c dominant negative Furin	CTCGATGCCATCGTTCAGAATGGAGACCACAATGC
4-20	cS2 Delta N679K+R681H	CCAGACACAGACAAAGAGCCACAGACGGGCC
4-21	nS2 Delta N679K+R681H	GGCCCGTCTGTGGCTCTTTGTCTGTGTCTGG
4-28	cS2 Omicron Y655H	GTCTGATCGGAGCCGAGCACGTGAACAATAGCTACG
4-29	nS2 Omicron Y655H	CGTAGCTATTGTTCACGTGCTCGGCTCCGATCAGAC
4-30	cS2 Delta H655Y	GTCTGATCGGAGCCGAGTACGTGAACAATAGCTACG
4-31	nS2 Delta H655Y	CGTAGCTATTGTTCACGTACTCGGCTCCGATCAGAC
4-34	cS2 Omicron K679N+H681R	CCAGACACAGACAAACAGCCGCAGACGGGCC
4-35	nS2 Omicron K679N+H681R	GGCCCGTCTGCGGCTGTTTGTCTGTGTCTGG

## Data Availability

Not applicable.
